# Tandem Mass Spectrometry in Untargeted Lipidomics: A Case Study of Peripheral Blood Mononuclear Cells

**DOI:** 10.3390/ijms252212077

**Published:** 2024-11-10

**Authors:** Giovanni Ventura, Mariachiara Bianco, Cosima Damiana Calvano, Ilario Losito, Tommaso R. I. Cataldi

**Affiliations:** Department of Chemistry, and Interdepartmental Research Center SMART, University of Bari Aldo Moro, Via Orabona 4, 70126 Bari, Italy; cosimadamiana.calvano@uniba.it (C.D.C.); ilario.losito@uniba.it (I.L.); tommaso.cataldi@uniba.it (T.R.I.C.)

**Keywords:** lipidomics, tandem MS, product ions, biomarker discovery, HILIC

## Abstract

Peripheral blood mononuclear cells (PBMCs), including lymphocytes, are important components of the human immune system. These cells contain a diverse array of lipids, primarily glycerophospholipids (GPs) and sphingolipids (SPs), which play essential roles in cellular structure, signaling, and programmed cell death. This study presents a detailed analysis of GP and SP profiles in human PBMC samples using tandem mass spectrometry (MS/MS). Hydrophilic interaction liquid chromatography (HILIC) and electrospray ionization (ESI) coupled with linear ion-trap MS/MS were employed to investigate the diagnostic fragmentation patterns that aided in determining regiochemistry in complex lipid extracts. Specifically, the study explored the fragmentation patterns of various lipid species, including phosphatidylcholines (PCs), phosphatidylethanolamines (PEs), their plasmalogen and lyso forms, phosphatidylserines (PSs), phosphatidylinositols (PIs), phosphatidylglycerols (PGs), sphingomyelins (SMs), and dihexosylceramides (Hex_2_Cer). Our comprehensive analysis led to the characterization of over 200 distinct lipid species, significantly expanding our understanding of PBMC lipidome complexity. A freely available spreadsheet tool for simulating MS/MS spectra of GPs is provided, enhancing the accessibility and reproducibility of this research. This study advances our knowledge of PBMC lipidomes and establishes a robust analytical framework for future investigations in lipidomics.

## 1. Introduction

Lipid MAPS [1], a consortium focused on the study of lipid metabolites and pathways strategies, has provided the most recent definition of the term “lipids” as a vast and heterogeneous class of hydrophobic or amphipathic small molecules that may originate entirely or partially from carbanion-based condensations of thioesters and/or carbocation-based condensations of isoprene units. For simplification, the Lipid MAPS consortium has classified lipids into eight categories: fatty acyls (FAs), glycerolipids (GLs), glycerophospholipids (GPs), sphingolipids (SPs), sterol lipids (STs), prenol lipids (PRs), saccharolipids (SLs), and polyketides (PKs). GPs are a large group of lipids that include at least one glycerol molecule esterified by a phosphate group at the *sn*-3 position and one or two acyl chains at the *sn*-1 and/or *sn*-2 positions. GPs are key components of cellular membranes and are involved in metabolism and signalling. The complexity of GP species arises from over 10 classes, several subclasses, and various individual molecular species with different fatty acyl chain structures. SPs are another category of cellular lipids, containing long-chained sphingoid bases as their core structures. The polar moiety linked to the hydroxyl group at position C_1_ of the sphingoid base determines the SP classes [2]. The variety of lipids in an organism is further increased due to isomers differing in double bond location, regiochemistry and stereochemistry. Accordingly, mammalian cells may contain more than 1000 lipid species [3].

It is well recognized that lipids play many essential roles in life [4]. For instance, they serve as building blocks in biological organisms and act as hydrophobic barriers in cellular membranes, enabling cellular compartmentalization. Furthermore, these compounds facilitate transmembrane protein function and signal transduction [5]. They also provide energy storage and supplementation for biological processes. Additionally, lipids are crucial regulators of metabolism and cellular actions, such as growth, differentiation, and apoptosis [6,7,8]. Moreover, numerous studies have shown that lipids are involved in various diseases, including tumorigenesis [9], atherosclerosis [10], diabetes [11], and Alzheimer’s disease [12].

Lipidomics aims to study the structure and function of the lipidome and its interactions with other lipids, proteins, and metabolites, searching for specific biomarkers for risk assessment and disease diagnosis on a large scale [13,14]. It was only in the early 2000s that Spener et al. described lipids as *terra incognita* [15] compared with proteins and metabolites. Over the last two decades, lipidomics has gained great significance, becoming one of the most important branches of omics and a very active research field. In 2011, Yang and Han [16] emphasized the importance of lipidomics by stating that “*any perturbation of a biological system is expected to alter the abundance and/or composition of the lipid pool*”, highlighting its relevance in biochemical and clinical fields. Interest in lipid characterization and quantification has also expanded to bioenergetics, nutrition, and the foodstuff areas.

The structural diversity of lipids presents a significant challenge in analytical chemistry [17,18,19]. However, advancements in mass spectrometry (MS) over the past two decades have greatly increased the field of lipidomics. MS/MS studies are essential for distinguishing between isobaric and/or isomeric lipid species [20,21]. Fragmentation analyses provide detailed structural information, allowing us to determine the composition of lipids, including the polar head group, fatty acyl chain length and unsaturation degree, and in some cases the position of double bonds [22,23,24,25]. Additionally, MS/MS can validate chromatography data, which is crucial in lipidomics.

The primary separation mechanisms exploited in lipidomic studies include hydrophilic interaction liquid chromatography (HILIC) and reversed-phase liquid chromatography (RPLC) [18,26,27,28,29,30]. HILIC separates lipids based on their polar head groups, allowing lipids from the same class to elute together. In contrast, RPLC separates lipids based on their fatty acyl chain compositions, often leading to the co-elution of lipids from different classes with similar acyl chains. In this context, MS/MS analyses are fundamental for identifying specific elution bands of lipids and distinguishing between isobaric or isomeric species, especially in clinical, medical, and biological research, where specific lipids may serve as biomarkers.

To highlight the critical role of MS/MS analysis in distinguishing between isomeric species and discuss potential artefacts that may occur during the process, we focused on the regiochemical characterization of the GPs identified in a study on the phospholipidome of peripheral blood mononuclear cells (PBMCs) aimed at discovering autism biomarkers [31]. In the present work, putative identifications accomplished by HILIC-ESI-MS retention times and accurate *m*/*z* values provided by Fourier transform MS (FTMS) using an orbital trap were corroborated by tandem MS (MS/MS) analyses with a linear ion trap (LIT) instrument and reported and discussed.

## 2. Results and Discussion

### 2.1. Glycerophospholipids of Peripheral Blood Mononuclear Cells

PBMCs are a diverse group of immune cells found in the blood, including lymphocytes (T cells, B cells, and natural killer cells), monocytes, and dendritic cells. PBMCs are formed in the bone marrow and are crucial in immune surveillance, response to infection, and inflammation, making them important in research for understanding immune-related diseases [32].

GPs play crucial roles in biological systems, serving as fundamental components of cell membranes and forming the basic structural framework that surrounds cells and organelles. Head groups in the *sn*-3 position can include various polar moieties, such as choline, ethanolamine, serine, or inositol, resulting in phosphatidylcholines (PCs), phosphatidylethanolamines (PEs), phosphatidylserines (PSs), and phosphatidylinositols (PIs), respectiverly, among others. The unique head groups of GPs contribute to the diversity of membrane properties and functions, influencing membrane fluidity, curvature, and the ability to interact with proteins and other molecules. Moreover, GPs are not static components; they participate dynamically in cellular signalling pathways. For example, PSs, typically located on the inner leaflet of the plasma membrane, play a role in apoptosis by flipping to the outer leaflet as an “eat me” signal for phagocytes [33]. In our study, the identification and characterization of GPs were performed by a linear ion trap MS instrument thanks to its ability to generate informative fragmentation patterns by collision-induced dissociation (CID) [34,35,36,37,38,39], which are crucial for deducing the composition of fatty acid chains and their regiochemistry.

### 2.2. Characterization of Nitrogen-Containing GPs in Extracts of PBMC Samples

**Figure 1:**
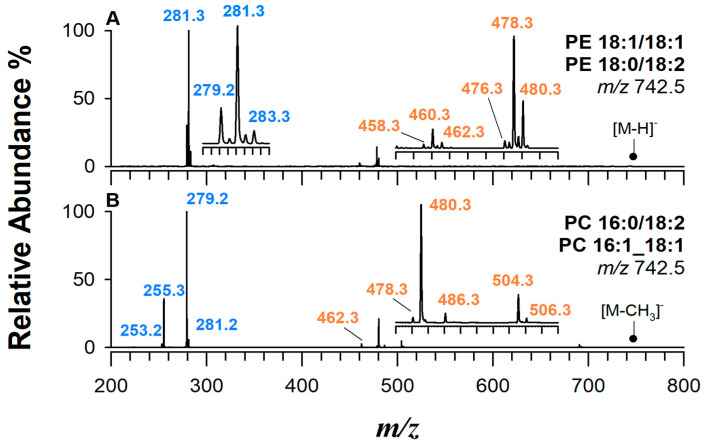
Tandem MS spectra by ESI(-)CID of isomeric species at *m*/*z* 742.5. (**A**) PE 18:1/18:1 and PE 18:0/18:2 and (**B**) demethylated PCs recognized as dimethyl-phosphoethanolamines (DMPEs) 16:0/18:2 and 16:1_18:1. The lipidomics analyses were performed on a sample extract of PBMCs.

The second most abundant phospholipids in mammalian membranes, accounting for approximately 15–25% of total PLs [40], are PEs, which contain an ethanolamine molecule attached to the phosphate group. While HILIC provides excellent separation of PLs due to its ability to exploit the hydrophilic properties of phospholipid head groups as shown in Figure S1, the coelution of isomeric species within the same lipid class is a common challenge and needs to be faced. An example of a composed tandem MS spectrum generated by two isomeric PEs is shown in Figure 1A.

Figure 1A shows the CID spectrum upon ESI in negative ion mode of a precursor ion at *m*/*z* 742.5 due to a PE 36:2. Three groups of peaks can be observed due to fatty acyl chain (FAC) ions and neutral losses of acyl chains both as ketenes (KEs) and neutral carboxylic acids (FAs), being acyl chains lost preferentially as KEs rather than FAs [41]. The prevalence of FA losses as KE is a common property observed in all GPs with gas-phase basicity.

The prominent peaks at *m*/*z* 281.3 accompanied by less intense signals at *m*/*z* 283.3 and *m*/*z* 279.2 correspond to carboxylate ions of FACs 18:1, 18:0, and 18:2, respectively. The other two clusters at *m*/*z* 480.3, 478.3, and 476.3 and *m*/*z* 462.3, 460.3, and 458.3 result from the neutral losses of FACs 18:2, 18:1, and 18:0 as KEs and FAs, respectively. While the generation of a relatively intense FAC 18:1 at *m*/*z* 281.3 and the detection of an intense peak due to 18:1 KE loss, at *m*/*z* 478.3, imply the occurrence of a PE 18:1/18:1, the other two carboxylate anions suggest the presence of another PE including 18:0 and 18:2 as FACs. In CID regimes, PEs preferentially lose FACs as KEs from the *sn*-2 position [36]. Thus, the higher intensity of the ion at *m*/*z* 480.3 ([M-H-KE 18:2]^−^) compared with *m*/*z* 476.3 ([M-H-KE 18:0]^−^) indicates that the less abundant species is a PE 18:0/18:2. To simulate MS/MS spectra in negative ion mode, a spreadsheet document was developed (*vide infra*). The structure of this spreadsheet, designed to perform calculations that mimic the fragmentation process, will be introduced in Section 2.7, along with a step-by-step guide on how to use this tool. An example of a simulated spectrum for deprotonated PE 18:0/18:2 can be found in the PE tab of the spreadsheet (Supplementary Information).

Another aspect of the analysis of PLs mixtures is the generation of artefacts due to the analysis conditions. However, this is not always a problem, and in some cases, these artefacts can be leveraged for in-depth characterization purposes. Indeed, PCs are structurally composed of a choline head group linked to the glycerol backbone through a phosphodiester bond. PCs are usually detected in positive ion mode due to the positively charged quaternary amine in the choline head group. However, the MS/MS spectra of protonated PC species do not provide information about their regiochemistry. To determine the regioisomeric structure, it is necessary to fragment cation adducts such as [M+Li]^+^ or [M+Na]^+^ [38]. However, managing the formation of these adducts can be problematic. For example, when using sodium, isobarism may complicate matters as there can be an overlap between ions like [PC X:Y+Na]^+^ and [PC (X+2):(Y+3)+H]^+^, unless very high mass resolution is available. For instance, the accurate *m*/*z* values for [PC 36:0+Na]^+^ and [PC 38:3+H]^+^ are 812.6140 and 812.6164, respectively. Due to these challenges, regioisomeric assignments in complex samples are typically made using negative polarity.

In negative ion mode, those GPs exhibit a preference for ionization by forming adducts with anions present in the mobile phases, such as the formate ion (HCOO^−^). Tandem mass spectrometry analysis of PC species in the form of formate adducts ([M+HCOO]^−^, where M represents the zwitterionic form of a PC) does not provide conclusive information regarding the acyl chain composition and regiochemistry (*sn*-1 vs. *sn*-2 positioning) of the PC molecules. This is because the primary fragmentation of these [M+HCOO]^−^ ions leads to the formation of demethylated PC ions ([M-CH_3_]^−^), which do not directly yield regiochemical information [31]. However, the subsequent fragmentation of the [M-CH_3_]^−^ ions can provide a more informative fragmentation pattern that can be used to infer the regiochemistry of the acyl chains [42,43]. Alternatively, CH_3_^+^ removal can occur directly within the ESI source by tuning the potentials [44], a process known as in-source-induced dissociation (SID), thereby eliminating the need for MS^3^ spectra for regiochemical identification.

Interestingly, [M-CH_3_]^−^ ions are gas-phase-formed deprotonated dimethylphosphoethanolamines (DMPEs), and it is worth noting that DMPEs are also biosynthetic intermediates in the synthesis of PCs; thus, they might be found in biological samples. They are important in yeast and have also been found in mouse liver [45]. It is essential to highlight that DMPEs are isomeric with PEs that have two additional carbon atoms in their sum composition. While artefacts originating from PCs can provide direct regiochemical information, these ions may be easily confused by DMPEs and PEs, regardless of MS resolution, if no preliminary chromatographic separation is accomplished, like in *shotgun* approaches, or when RPLC is employed, since the latter is unable to separate PLs based on their polar head. HILIC retention times, combined with a comparison of positive and negative ion mode spectra, provide the most reliable method for accurate lipid identification. Furthermore, tandem MS spectra offer a powerful tool to differentiate between PEs and DMPEs, whether they are naturally occurring or generated during gas-phase reactions. This differentiation is possible due to the distinct FAC profiles and diagnostic signals produced by these lipid classes. Figure 1B presents the MS/MS spectrum of the ion at *m*/*z* 742.5, which corresponds to a demethylated deprotonated PC 34:2. As evident, this is a structural isomer of the examined deprotonated PE 36:2. Two major peaks were observed at *m*/*z* 279.2 and *m*/*z* 255.3, revealing the presence of FACs 18:2 and 16:0, respectively. The relative intensities of *m*/*z* 480.3 and *m*/*z* 504.3, resulting from the loss of FACs as KE, suggested that the species can be identified as PC 16:0/18:2. Interestingly, the carboxylate ions at *m*/*z* 253.2 and 281.2, along with the less prominent ions resulting from the loss of the corresponding acyl chains as KE, account for the presence of PC 16:1_18:1 in the sample. In cases where the relative intensities of the ions are not significantly different, the regioisomeric assignment becomes less reliable. This often occurs when less abundant species co-elute during HILIC separation, potentially representing a mixture of different regioisomers. Furthermore, intensity differences can be obscured by the co-elution of a more abundant isomer, as was the case in this study. For these less abundant species, it is prudent to refrain from making a definitive regioisomeric identification. In such cases, it is more appropriate to report only the overall fatty acid-level composition, without attempting to assign the specific regiochemistry.

As highlighted earlier, distinguishing between the isomeric species corresponding to deprotonated forms of PE 36:2 and of demethylated PC 34:2 using FTMS alone, if PE 36:2 and PC34:2 co-elute significantly, as it might happen during RPLC separations, is challenging. The presence of less abundant ions (e.g., *m*/*z* 242.1 and 224.1 for PCs and *m*/*z* 196.0 and 214.1 for PEs; see Figure S2), which are well documented in the literature [39,46], aids in identifying the lipid class. However, distinguishing between the MS/MS spectra of a demethylated deprotonated PC and a deprotonated DMPE remains impossible, and employing HILIC separations is mandatory as it resolves these ambiguities by providing orthogonal information through retention time. Indeed, DMPEs originally present in a matrix are expected to exhibit different retention compared with PCs under HILIC conditions. Table S1 summarizes the regiochemistry of PEs and PCs found in the PBMC sample.

### 2.3. Plasmalogens in Extracts of PBMC Samples

Plasmalogens or ether lipids are characterized by the presence of either an ether or a vinyl ether bond at the *sn*-1 position of the glycerol backbone, rather than the more common ester bond found in most GPs. This structural variation gives rise to plasmanyl (1-O-alkyl-2-acyl-sn-glycerol-3-phosphate) and plasmenyl (1-O-alkenyl-2-acyl-sn-glycerol-3-phosphate) lipids, which are designated with the prefix “O-” and “P-”, respectively. These lipid subclasses exhibit different biochemical and biophysical properties compared with their more common counterparts. Indeed, structural unique features contribute to their specific roles in various cellular processes and have potential roles as biomarkers in several diseases [47,48]. Often, without distinguishing between ether-linked alkyl or alkenyl species, the letter -O is simply added after the lipid class abbreviation to indicate that the species in question belongs to either of the two classes. Ether lipids are found in a variety of lipid classes, including PCs, PEs, phosphatidic acids (PAs), phosphatidyl glycerols (PGs), triglycerides (TAGs), and cardiolipins (CLs) [49].

The MS/MS spectra of ether-linked PCs and PEs are very similar to those of the corresponding diacyl forms. The main difference is that the fatty alcohol is lost much less easily, resulting in the signals related to its loss and the alkoxylates of the *sn*-1 chains being either absent or relatively less abundant. Like diacyl forms, PC O- (observed as demethylated species) and PE O- (observed as deprotonated species) might be structural isomers. While distinguishing between these species is relatively straightforward by searching for diagnostic product ions and/or using HILIC retention times (see Figures S1 and S2), distinguishing between alkyl or alkenyl forms is notably more challenging.

**Figure 2:**
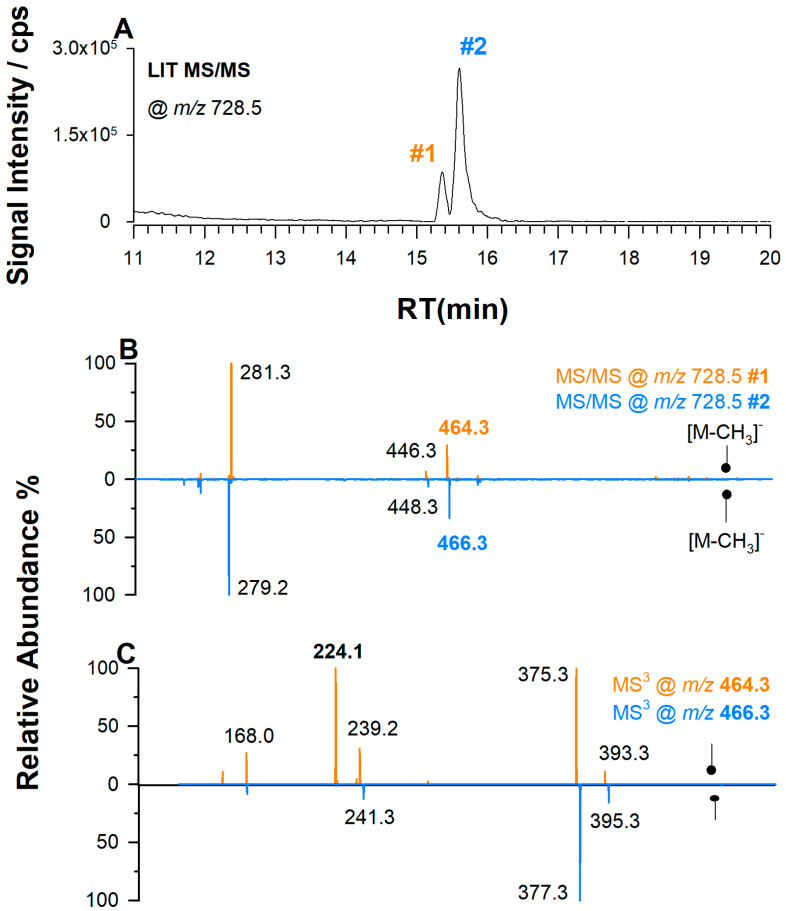
(**A**) XIC of *m*/*z* 728.5 reveals the occurrence of two PC plasmalogens labeled 1 and 2. (**B**) Tandem MS spectra of both chromatographic peaks 1 and 2. (**C**) MS^3^ spectra of the precursor ions at *m*/*z* 464.3 and 466.3 of peaks 1 and 2, respectively. The product ion at *m*/*z* 224.1 observed for the chromatographic peak 1 confirms the presence of two isomeric species: PC P-16:0/18:1 (peak 1) and PC O-16:0/18:2 (peak 2).

To distinguish between alkyl and alkenyl species of PCs, two approaches can be employed: (1) MS/MS analysis in positive ion mode, which allows for direct recognition of these species [50], and (2) MS/MS analysis in negative ion mode, where the difference in product ion intensities at *m*/*z* 224.1 in the MS^3^ spectrum can be used to discriminate between the two species. This ion, common to PCs, is generated from the loss of both *sn*-1 and *sn*-2 side chains and has a structure as reported in Figure S2 [38,45]. The product ion at *m*/*z* 224.1 appears as the base peak in the spectrum derived from PC P- species, while it is negligible in the spectrum derived from PC O- ones [51]. Under the adopted experimental conditions used in this study, a PC O- and a PC P- having the same sum composition at *m*/*z* 728.5 exhibited two nearly separated peaks (i.e., peaks 1 and 2), as shown in plot A of Figure 2.

The main differences in MS/MS spectra depicted in Figure 2B are related to the peak signals at *m*/*z* 281.3 due to a FAC 18:1 under peak 1 and at *m*/*z* 279.2 of a FAC 18:2 under peak 2, along with the peaks generated after their loss as KEs, resulting in the gas-phase formation of LPC O- at *m*/*z* 464.3 and *m*/*z* 466.3, respectively. As mentioned above, the signals related to the loss of the *sn*-1 chain are not observed. MS^3^ analysis confirms that peak 1 corresponds to PC P-16:0/18:1, whereas peak 2 corresponds to PC O-16:0/18:2 due to the differing intensity of the ion at *m*/*z* 224.1. Note that the vinyl–ether bond is not counted as a double bond within the ether-linked chain in the adopted nomenclature system [52]. It should be noted that signals of the corresponding fatty alcohols are also observed in MS^3^ spectra at *m*/*z* 239.2 and *m*/*z* 241.3.

**Figure 3:**
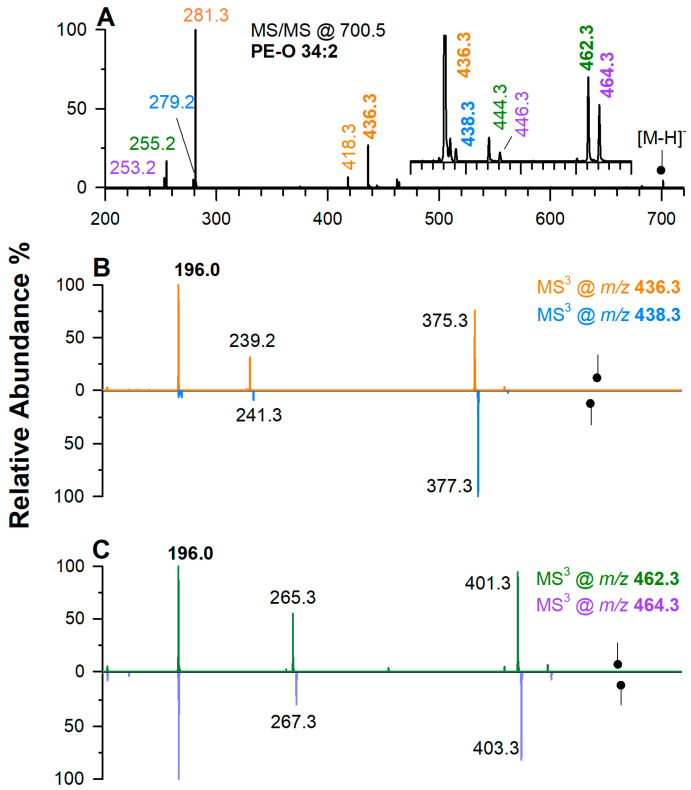
(**A**) Tandem MS spectrum by ESI(-)CID at *m*/*z* 700.5 of PE O-34:2. (**B**) CID-MS^3^ spectra of LP O-16:0 at *m*/*z* 438.3 (blue trace) and LPE P-16:0 at *m*/*z* 436.3 (orange trace). (**C**) CID-MS^3^ spectra of LPE P-18:1 at *m*/*z* 462.3 (green trace) and LPE P-18:0 at *m*/*z* 464.3 (violet trace).

Likewise, discrimination between PE O- and PE P- with a sum composition of 34:2 can be achieved by observing the MS^3^ spectra of LPE O- generated in the gas phase. Although the chromatographic peaks were not well separated as it happened for ether-PCs, they were still distinguishable in the MS^n^ domain. The MS/MS spectrum of PE O-34:2 at *m*/*z* 700.528 is shown in Figure 3A.

As evidenced by signals related to FACs 18:1 (*m*/*z* 281.3), 16:0 (*m*/*z* 255.2), 16:1 (*m*/*z* 253.2), and 18:2 (*m*/*z* 279.2), such a spectrum is generated by the combination of several contributions. In addition, four interesting signals are observed and highlighted in bold in different colors, which can be easily ascribed to LPE O- 16:1 (*m*/*z* 436.3), 18:2 (*m*/*z* 462.3), 18:1 (*m*/*z* 464.3), and 16:0 (*m*/*z* 438.3). Combining data of chromatographic retention and precursor leads to a PE-O 34:2 as the likely combination, which leads to the following arrangements: 18:2_16:0, 18:1_16:1, 16:1_18:1, and 16:0_18:2. The MS^3^ spectra of the lyso PE-O species generated in the gas phase are displayed using the same colors in Figure 3B,C. Among the four PE plasmalogens, the blue spectrum is due to a PE O-16:0/18:2 because the absence of double bonds on the *sn*-1 chain is confirmed by the low intensity of the ion at *m*/*z* 196.0. Conversely, the same product ion is the base peak in all other spectra, which are recognized as due to PE P-18:1/16:0, P-18:0/16:1, and P-16:0/18:1. In Table S2 are summarized all PE-O and PC-O found in the PBMC sample. Note that a distinction between plasmanyl and plasmenyl PLs is not always provided.

### 2.4. Characterization of Lyso-Species Contained in Extracts of PBMC Samples

Lysophospholipids (LGPs) are modified GPs where one fatty acid chain has been removed, typically by phospholipase enzymes or degradation processes. These molecules are important in cell signalling and can be analyzed using MS/MS in negative ion mode to determine their structural features. In the HILIC separation of PBMC extracts, LGPs like lysophosphatidylcholines (LPCs) and lysophosphatidylethanolamines (LPEs) show two peaks, corresponding to different regiochemistries. For example, LPE 20:4 exhibits distinct MS/MS spectra, with the carboxylate ion of FA 20:4 at *m*/*z* 303.3 as the base peak (see Figure S3). For LPEs, the first chromatographic peak corresponds to species with the fatty acid at the *sn*-2 position. This distinction can be further confirmed by examining the relative intensities of product ions at *m*/*z* 196.0 and *m*/*z* 214.1 in the MS/MS spectra: the latter is more intense for the first peak, while the opposite occurs for the second [39] (see Figure S2). Similarly, LPCs also display two chromatographic peaks for a given *m*/*z* ratio, where specific ions, such as *m*/*z* 224.1 and *m*/*z* 242.1, serve as markers for stereochemistry, helping to distinguish the position of the remaining FAC: the peak signal at *m*/*z* 224.1 is more intense for species with the residual chain at the *sn*-1 position (see plots B and C of Figure S4) [39]. The supporting spreadsheet document (*vide infra*) allows also for the simulation of the MS/MS spectra of the two regioisomers (Supplementary Information).

**Figure 4:**
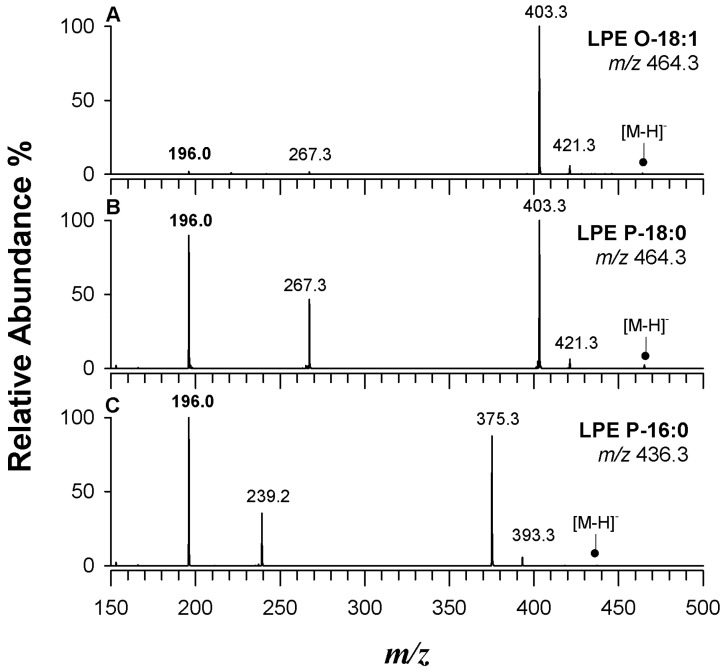
Tandem MS spectra of (**A**) LPE O-18:1 at *m*/*z* 464.3, (**B**) LPE P-18:0 at *m*/*z* 464.3, and (**C**) LPE P-16:0 at *m*/*z* 436.3.

Remarkably, the residual chain in ether lipids is typically at the *sn*-1 position. However, the regiochemistry of LPE O- and LPC O- can be confirmed by CID tandem MS analysis [53]. Figure 4 shows the MS/MS spectra of (A) LPE O-18:1, (B) LPE P-18:0, and (C) LPE P-16:0, where the signals at *m*/*z* 239.2 and *m*/*z* 267.3 correspond to deprotonated alcohols, namely, 16:1 and 18:1, respectively.

The ESI(-)CID-MS/MS spectrum of LPE O- is defined by the loss of ethanolamine (C_2_H_7_NO, 61.05 Da), producing ions at *m*/*z* 403.3 and *m*/*z* 375.3. The absence of a peak at *m*/*z* 214.1 indicates the acyl chain is not at the *sn*-2 position, typical of *sn*-1 isomers. The intensity of the peak at *m*/*z* 196.0 varies depending on the plasmalogen type, being prominent in plasmenyl LPE but minimal in plasmanyl LPE. For plasmanyl LPC, the lack of a peak at *m*/*z* 242.1 supports its regiochemical designation.

In Figure S5, the MS/MS spectra of the LPC P-16:0 and its structural isomer LPC O-16:1, fragmented as demethylated negative ions at *m*/*z* 464.3, are reported. Peaks at [M-89.0]^−^ and [M-89.0+18.0]^−^ are noted due to the loss of the demethylated choline head group [54]. Again, the ion at *m*/*z* 224.1 is prominent in LPC P-16:0 but negligible in LPC O-16:1. Additionally, an ion at *m*/*z* 239.3, corresponding to a deprotonated fatty alcohol 16:1, was observed in LPC P-16:0.

### 2.5. Acidic Glycerophospholipids in Extracts of PBMC Sample

PAs are the simplest class of GPs, consisting of a phosphate group attached to a diacylglycerol moiety. PAs also serve as key intermediates in the biosynthesis of other important lipid molecules such as PCs and PEs through the CDP-diacylglycerol pathway, contributing to the dynamic regulation of membrane composition. Beyond its role as a membrane constituent, PAs are potent signalling molecules involved in various cellular processes, including cell growth, proliferation, and intracellular trafficking [55].

Despite the relatively low content of PAs in the investigated PBMC sample, the fragmentation route of a PA [56] can be used as a model to explain the fragmentation of more common GPs. Unlike PEs and PCs, each FAC is primarily lost as a FA during fragmentation, rather than as KE, and also, in this case, the signals due to the losses of the acyl chains, both as FAs and KEs, are more intense from the *sn*-2 position [56]. This fragmentation pattern is also observed in the MS/MS spectra of other GPs with a gas-phase acidic character, such as PGs, PIs, and PSs. Also in this case, the intensity of FAC ions is not reliable for definitive assignment due to potential modifications, such as further fragmentation of unsaturated chains, which can change the relative intensities of FA ions and make the interpretation less straightforward. For instance, the deprotonated polyunsaturated FA 22:6 ([C_22_H_31_O_2_]^−^) at *m*/*z* 327.233 exhibits lower intensity because of the additional loss of CO_2_, resulting in an ion at *m*/*z* 283.243 ([C_21_H_31_]^−^) that is isobaric ([C_18_H_35_O_2_]^−^) with stearic acid (18:0) at *m*/*z* 283.264 [57]. Therefore, special attention is required because the gas-phase formation of PAs from other acidic lipids is rather common.

**Figure 5:**
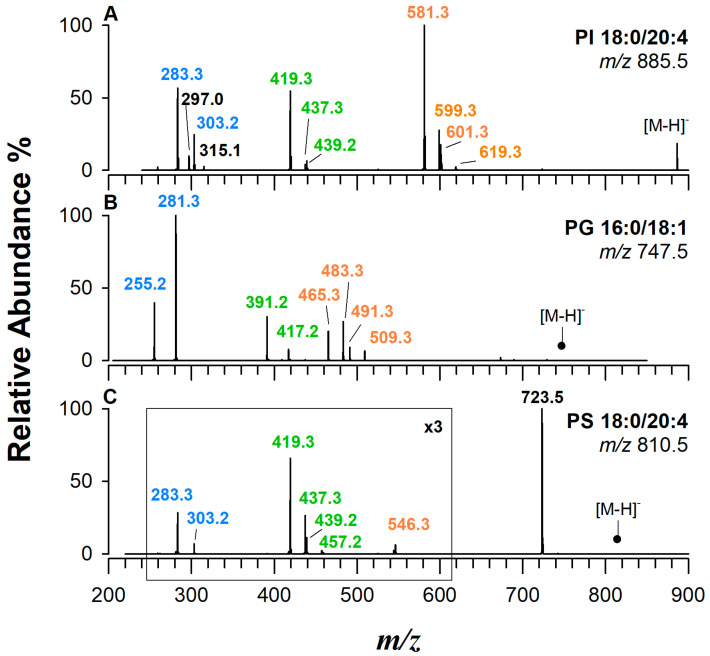
MS/MS spectra in negative ESI polarity using a linear ion trap instrument: (**A**) PI 18:0/20:4, (**B**) PG 16:0/18:1, and (**C**) PS 18:0/20:4. Product ions resulting from the FAC anions are depicted in blue, ions resulting from the loss of FA from the intact precursor are shown in orange, and ions formed with the same process from the gas-phase-formed PA are displayed in green.

Figure 5 shows representative CID-MS/MS spectra of PI at *m*/*z* 885.5 (A), PG at *m*/*z* 747.5 (B), and PS at *m*/*z* 810.5 (C) in the analyzed PBMC sample. Product ions resulting from the FAC anions are depicted in blue, while ions resulting from the loss of FA from the intact precursor are shown in orange. Additionally, ions formed with the same process from the gas-phase-formed PA are displayed in green.

In PIs, a molecule of inositol is linked to the phosphate group. PIs play a central role in cell signalling and membrane function. These lipids are key precursors for the synthesis of several critical signalling molecules, including phosphoinositides [58]. PIs are crucial components of cell membranes, contributing to their structure, integrity, and fluidity. In addition to the common neutral losses observed for PAs, such as the loss of acyl chains as KE and FA, PIs exhibit a series of interesting head-related signals, such as those at *m*/*z* 315.0 [C_9_H_16_O_10_P]^−^, *m*/*z* 297.0 [C_9_H_14_O_9_P]^−^, and *m*/*z* 241.0 [C_6_H_10_O_8_P]^−^ (see Figure S2), offering valuable information for the identification of this lipid class [59]. To illustrate the fragmentation process of PIs, Figure 5A shows the CID-MS/MS spectrum of the most intense peak detected within the PI chromatographic band, i.e., PI 38:4 at *m*/*z* 885.5. The signals observed at *m*/*z* 599.3 and *m*/*z* 581.3, as well as at *m*/*z* 619.3 and *m*/*z* 601.3, are generated by the acyl chains losses as KE or FA from the *sn*-2 and *sn*-1 positions, respectively. By analyzing their relative intensities, it becomes straightforward to assign the regiochemistry of the species as PI 18:0/20:4. It is intriguing to observe that signals that are typically observed in the MS/MS spectrum of a PA with the same sum composition, such as the signals at *m*/*z* 439.2 and *m*/*z* 419.3, are also detected in the MS/MS spectrum of a PI. This is because these signals are produced by the loss of inositol, a process that generates gas-phase PA, followed by the loss of acyl chains. These processes are common for PGs and PSs.

PGs, whose head group consists of a glycerol molecule, are essential phospholipids found in cell membranes and recognized as precursors for mitochondrial cardiolipins [60]. Figure 5B shows the MS/MS spectrum of the precursor ion at *m*/*z* 747.5 due to a PG 34:1. The presence of FA 16:0 and FA 18:1 was confirmed by the occurrence of signals at *m*/*z* 255.2 and 281.3, respectively. Furthermore, signals related to their losses both as FA (*m*/*z* 491.3 and 465.3) and as KE (*m*/*z* 509.3 and 483.3) were detected. As in the case of PI, the product ions at *m*/*z* 417.2 and *m*/*z* 391.2 can formally be explained as resulting from the neutral loss of C_3_H_6_O_2_ (74.0 Da; see Figure S2) from *m*/*z* 491.3 and 465.3, respectively, resulting in the gas-phase formation of a dehydrated lyso-PA [37]. Since the detachment of the chain from the *sn*-2 position is favored, the established regiochemistry is PG 16:0/18:1. PGs are structural isomers of bis(monoacylglycero)phosphate (BMP) species, with distinct properties and biological functions. Distinguishing PG from BMP using MS/MS spectra in negative ion mode is not possible, as identical spectra are produced. However, characterization of these two distinct PL classes can be achieved in positive ion mode fragmenting [M+H]^+^, [M+Na]^+^ [61], or [M+NH_4_]^+^ [62] ions. Nonetheless, both species ionize more efficiently in negative ion mode. For their recognition, an interesting approach was suggested by Wang et al. [63] concerning the phosphate methylation of PG and BMP. In addition, as reported by Cai et al. [64], phosphate methylation is a powerful approach to improve the ionization efficiency of anionic phospholipids in positive ion mode by eliminating the negative charge of the phosphate group. Indeed, distinctive MS/MS spectra in positive ionization mode can be obtained, yet were not employed in this study.

The polar head group of PSs plays a crucial role in their biological functions, primarily due to the unique properties conferred by the serine moiety. The overall negative charge of the PS head is critical for its involvement in cellular processes such as cell signalling, and recognition, and serving as a binding site for various proteins linked to signalling cascades and membrane trafficking [3]. In our experimental conditions, PSs elute in a broad chromatographic band between 10 and 13 min and ionize in negative ion mode, mainly as deprotonated species. The MS/MS spectrum of PS 38:4 (*m*/*z* 810.5) is shown in Figure 5C. The fragmentation pattern is dominated by the product ion at *m*/*z* 723.5, which is due to the neutral loss of dehydroserine (C_3_H_5_NO_2_, 87.0 Da; see Figure S2) and is often employed to confirm attribution. The loss of serine and FA 20:4 leads to the formation of ions at *m*/*z* 419.3 and *m*/*z* 437.3, while the loss of serine and FA 18:0 leads to the formation of ions at *m*/*z* 439.2 and *m*/*z* 457.2. In both cases, these ions are the same as those found in PAs with the same sum composition, which is also common to PI species. The intensity ratio of both product ions (i.e., *m*/*z* 419.3 > *m*/*z* 439.2 or *m*/*z* 437.3 > *m*/*z* 457.2) can be used to determine the regiochemistry of the PS, i.e., PS 18:0/20:4. Both carboxylate fatty acids 18:0 and 20:4 can be seen at *m*/*z* 283.3 and *m*/*z* 303.2, respectively. Since PCs can form adducts with acetate anions in negative polarity, thus becoming isomeric with PSs, tandem MS spectra of PSs are particularly useful to distinguish these PLs because their spectra are completely different. Table S3 summarizes the regiochemistry of PGs, PSs, and PIs identified in a sample extract of human PBMCs.

### 2.6. Sphingolipids in Extracts of PBMC Samples

**Figure 6:**
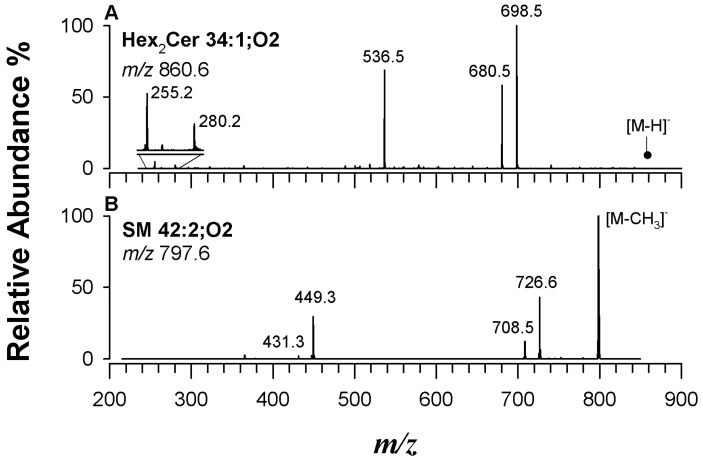
Tandem MS spectra by CID as deprotonated species of (**A**) Hex_2_Cer 34:1;O2, identified as Hex_2_Cer d18:1;O2/16:0 at *m*/*z* 860.6 with its 18:1;O2 sphingoid base and a 16-carbon saturated fatty acid (palmitic acid) attached to the ceramide, and (**B**) SM 42:2;O2 recognized as SM 18:1;O2/18:0 at *m*/*z* 797.6.

Sphingolipids (SLs) occur in the plasma membranes of all eukaryotic cells. They are composed of a sphingoid base backbone, which differentiates them from other lipid classes [2]. SLs are involved in cell signalling, membrane structure, and cell–cell communication and have been also implicated in various diseases and conditions, including neurodegenerative disorders, cancer, and metabolic disorders. The basic structure of a sphingolipid consists of a long-chain amino alcohol called a sphingoid base, which is linked to a fatty acid through an amide bond. Variation in the polar head connected to the sphingoid base gives rise to different sphingolipid species. For example, dihexosyl ceramides (Hex_2_Cer) consist of two hexose sugars and a ceramide (see Figure S6). Ceramides serve as a precursor for the synthesis of other complex sphingolipids, including more complex glycosphingolipids. When analyzed in negative ion mode, Hex_2_Cer (M) ionizes as the deprotonated form ([M-H]^−^) or as formate ([M+HCOO]^−^) and chlorinate ([M+Cl]^−^) adducts. Upon CID fragmentation of chlorinated and formate adducts, no information about regiochemistry is attained, since the most prominent peak corresponds to the neutral loss of HCl or HCOOH, leading to the formation of deprotonated Hex_2_Cer [2]. On the contrary, the fragmentation pattern of deprotonated Hex_2_Cer provides more informative data, revealing the neutral loss of one (162.0 Da) or two sugar moieties (324.0 Da), followed by water loss (18.0 Da). As an example, the MS/MS spectrum of *m*/*z* 860.6 (Hex_2_Cer 34:1;O2) found in the extracts of the human PBMC sample is reported in Figure 6A. The nomenclature (Hex_2_Cer 34:1;O2) refers to a specific dihexosylceramide in which the first number (34) represents the sum of carbon atoms in the fatty acid chain and sphingoid base, the number (1) after the colon indicates the number of double bonds, and the second number (O2) after the semicolon and the oxygen symbol indicates O atoms in fatty acyl/alkyl residues. Note that the oxygen atom at position 1 of the ceramide is also included in this count.

Together with the previously mentioned signals (i.e., *m*/*z* 698.5, *m*/*z* 680.5, and *m*/*z* 536.5), additional signals from dehydrated C18-sphingid base ([C_18_H_34_NO]^−^) and FAC 16:0 were observed at *m*/*z* 280.2 and *m*/*z* 255.2, respectively. The formation of the latter derives from an exclusive rearrangement of the acyl chain [35,65]. In this study, a significant portion of the identified Hex_2_Cer species possessed a C18-sphingoid base with one unsaturation, as reported in Table S1, with Hex_2_Cer 42:1;O2, Hex_2_Cer 40:1;O2, Hex_2_Cer 42:2;O2, and Hex_2_Cer 34:1;O2 accounting for over 85% of all ceramides found [31].

Another important SL class is represented by sphingomyelins (SMs), which contain a phosphorylcholine head group (Figure S6) and are abundant in the myelin sheath that surrounds nerve cells. Like PCs, which they share the polar head group with, SMs also form demethylated adducts in negative ion mode. These adducts were exploited for regiochemical characterization as tandem mass spectra of [M+HCOO]^−^ ions did not provide useful information on the acyl chains. Figure 6B displays the MS/MS spectrum of the ion at *m*/*z* 797.6 of a demethylated deprotonated SM, which would correspond to a SM 42:2;O2 species. Two intense neutral losses of 71.0 Da and 89.0 Da were observed, corresponding to the neutral loss of dehydrated or as such *N*,*N*-dimethylethanolamine, respectively, characteristic of this lipid class, when demethylated deprotonated precursor ions were considered. For regiochemical characterization, an informative peak at *m*/*z* 449.3, corresponding to the loss of FAC as a KE, was identified. The neutral loss of 348.3 Da (i.e., 364.3–18.0 Da) confirmed the presence of a 24:1 fatty acyl chain lost as KE. This, in turn, indirectly indicated the presence of an 18-carbon sphingoid base, allowing SM 42:2;O2 to be annotated as SM 18:1;O2/24:1. All the identified species are listed in Table S4. Remarkably, SM 34:1;O2, SM 40:1;O2, and SM 42:2;O2 collectively contributed to over 75% of the total class area in the sample extract of PBMCs [31].

### 2.7. MS/MS Spectrum Simulation Tool

To support lipidomics studies as in the case of PBMCs, we developed a spreadsheet layout by Excel^TM^ to simulate MS/MS spectra in negative ion mode for various lipid classes, including demethylated PC, deprotonated PE, PI, PS, PA, PG, and lyso and plasmalogen forms. This tool is provided as supplementary material, with its functionality described below and summarized in Figure S7. The user begins by selecting the tab corresponding to the lipid class of interest, as illustrated in the PE example. Then, the composition of FACs is entered by the user into the designated fields (marked as Section 1 in Figure S7), with the number of carbon atoms input in the blue cells and the number of double bonds in the green cells, respectively. If the plasmalogen form is required, entering “AL” in the orange field will generate the corresponding spectrum. Note that in such cases, users must manually adjust relative intensities (*vide infra*). The MS/MS spectra of plasmanyl and plasmenyl LPC and LPE can also be generated. In two separate tabs (namely, LPC O and LPE O), users can specify “P” or “O” in the orange field to generate the tandem MS spectra of the two different forms, and the intensity of the discussed diagnostic ions will vary accordingly. A summary table (Section 2) automatically generates the *m*/*z* values of the expected ions, and the simulated spectrum is displayed in Section 3. Relative intensities, which users input based on their specific MS instrument, are entered in Section 4. Since relative intensities vary significantly across different instruments, universal values cannot be applied. The reported intensities are comparable to those we obtained from the analysis of standards under the conditions outlined in this article. Users are free to adjust the intensities after assessing the fragmentation behavior at a specific energy and under the fragmentation regime used.

By toggling checkboxes in Section 4, users can show or hide specific ions of interest. Diagnostic product ions for each lipid class are also listed (see Figure S2).

In MS analyses, consideration of the low mass cutoff associated with certain analyzers, especially ion traps, is essential as this limitation can significantly influence the observed spectral data [66]. The simulator is designed for flexibility across various analyzers, calculating exact *m*/*z* ratios and displaying values to the fourth decimal place. However, because it is not specifically optimized for the low mass cutoff typical of ion traps, ions in lower *m*/*z* ranges that would normally be undetected on such instruments may appear. While MS analyzers like Orbitrap and ToF generally detect reported diagnostic ions at low *m*/*z* without adjustments, ion trap instruments often require specific modifications to capture these ions effectively [67]. To address this variability and improve alignment between simulated and experimental spectra, users can set the minimum *m*/*z* value in the generated spectrum, enhancing the consistency between simulated data and experimental results.

This Excel routine can simulate various fragmentation patterns in negative ion mode for different lipid classes, providing a quick reference for lipidomics studies. Although existing software and online tools offer similar functionality, this freely available spreadsheet will be handy for those new to lipidomics.

Studying the lipidome is crucial, particularly in interesting compounds such as PBMCs. Recent research has highlighted a dysregulation of SL in the context of diabetes in children [68], as well as a disruption of PLs and SLs in obese patients with dysglycemia [69]. To the best of our knowledge, the modification of regiochemistry in the PBMC lipidome has not yet been correlated with any pathologies. We believe that a detailed examination of the PBMC lipidome could potentially provide new insights for diagnostic diseases and other conditions in the future, However, the primary aim of this article was to utilize this intriguing sample as a model to demonstrate the significance of MS/MS spectra in lipidomic characterization, providing simple tools to aid in this complex task.

## 3. Materials and Methods

### 3.1. Chemicals

LC-MS grade water, ACN, methanol (MeOH) and HPLC grade chloroform, formic acid, and ammonium formate were obtained from Merk (Milan, Italy). Standard solutions for negative calibrations were purchased from Thermo Scientific (Waltham, MA, USA).

### 3.2. Lipid Nomenclature

Lipids were named according to the Comprehensive Classification System for Lipids [70]. Accordingly, the symbol “/” was used when the regiochemistry was known. For example, PC 16:0/18:1 referred to phosphatidylcholine where the *sn*-1 FAC had 16 carbons and no double bonds, and the *sn*-2 FAC had 18 carbons with one double bond. Conversely, the symbol “_” was used to denote unknown regiochemistry. For example, PC 16:0_18:1 indicated that the two fatty acyl chains were 16:0 and 18:1, but their positions on the glycerol backbone (*sn*-1 or *sn*-2) were not specified. For SLs (e.g., SM 18:1;O2/24:1), the first part (18:1;O2) indicated the number of C atoms, DBs, and O atoms in the sphingoid base, respectively, while the numbers following the slash denoted the C atoms and DBs in the acyl chain. Note that the O count included the O atom at position 1 of the ceramide.

### 3.3. Lymphocyte Samples

QC samples [71], obtained as discussed in [31], were employed for those analyses. For the Bligh and Dyer protocol [72], approximately 2 × 10^6^ lymphocyte cells, obtained as discussed in [31], were dissolved in 400 μL of LC-MS-grade water and 1.5 mL of methanol/chloroform (2:1, *v*/*v*) were added to the solution and left for 1 h at room temperature. Then, 0.5 mL of chloroform was added, and the mixture was vortexed for 30 s. Finally, 0.5 mL of water was added, and the solution was shaken before being centrifuged for 10 min at 3000× *g*. The lower phase containing lipids was dried under nitrogen; the residue was dissolved in 100 μL of methanol. The use of PBMCs isolated from fresh whole blood for this study was approved by the Local Ethics Committee of Azienda Ospedaliera Universitaria (Bari, IT) (approval number 164, 11 November 2016).

### 3.4. Instrumentation and Operating Conditions

A Velos Pro mass spectrometer, equipped with a linear ion trap (Thermo Scientific, Waltham, MA, USA), was employed for MS and MS/MS detection after HILIC separations. The heated ESI interface parameters were as follows: sheath gas flow rate at 35 a.u., auxiliary gas flow rate at 5 a.u., spray voltage at 2.5 kV (negative polarity), capillary temperature at 350 °C, and S-lens RF level at 64.2 a.u. MS full-scan acquisitions were performed in negative ion mode within the *m*/*z* range of 300–1500. For targeted MS/MS and MS^3^ analyses, the normalized collision energy was set to 35% (with 100% corresponding to a 25 V excitation voltage) using a 1 *m*/*z* unit-wide isolation window centered on the target *m*/*z* ratio. A silica-phase Ascentis Express HILIC column (150 × 2.1 mm i.d., 2.7 μm particle size) equipped with an Ascentis Express HILIC security guard cartridge (50 × 2.1 mm i.d.) from Supelco (Bellefonte, PA, USA) was used for chromatographic separations at a flow rate of 0.3 mL/min at 25 °C. Five μL of the sample were manually injected. The binary elution program, using water with 2.5 mmol/L ammonium acetate (solvent A) and ACN (solvent B), both containing 0.1% (*v*/*v*) formic acid, was as follows: 0–5 min, linear gradient from 97 to 88% solvent B; 5–10 min, isocratic at 88% solvent B; 10–11 min, linear gradient from 88 to 81% solvent B; 11–20 min, linear gradient from 81 to 70% solvent B; 20–22 min, linear gradient from 70 to 50% solvent B; 22–28 min, isocratic at 50% solvent B; 28–30 min, return to the initial composition, followed by a 5 min equilibration time. It is worth noting that under the described chromatographic conditions, formate adducts were preferentially formed during ESI in negative polarity, despite acetate anions also being present in the mobile phase. LC-MS instrumentation control and initial data processing were performed using Xcalibur software 2.2 SP1.48 (Thermo Scientific).

## 4. Conclusions

This study emphasizes the important role of tandem MS analysis by CID in the field of untargeted lipidomics, particularly when distinguishing between closely related species in biological samples is mandatory. The approach described herein uses ESI in negative ion mode and tandem MS, following HILIC separation, to confirm lipid molecule identification, which reduces false positives in the molecular assignment. By reviewing and applying the fragmentation rules of various PLs, a significant number of lipid species occurring in the sample extracts of PBMCs is annotated. Using this approach, we were able to recognize the most abundant PLs in PBMCs and to determine their composition of fatty acid constituents. The results highlight that MS/MS spectra are often crucial for isomeric lipid species, such as plasmalogen forms by MS^3^ analyses in negative ion mode, or tandem MS to confirm putative assignment obtained from high-resolution MS data. To assist with data interpretation, a user-friendly freely available spreadsheet layout for MS/MS spectrum simulation is presented. Overall, our research provides a comprehensive characterization of complex lipid composition in PBMCs.

## Data Availability

The raw data supporting the conclusions of this article will be made available by the authors upon request.

## Supplementary Materials

The following supporting information can be downloaded at https://www.mdpi.com/article/10.3390/ijms252212077/s1.
