# Supplementary material for: Tandem Mass Spectrometry in Untargeted Lipidomics: A Case Study of Peripheral Blood Mononuclear Cells
Source: Int J Mol Sci. 2024 Nov 10;25(22):12077. doi: 10.3390/ijms252212077 (PMC11593930; doi:10.3390/ijms252212077)
Supplement: Supplementary file 1 [file ijms-25-12077-s001.zip › Supporting 1.pdf]

International Journal of *Molecular Sciences*

MDPI

Supporting Information

Tandem mass spectrometry in untargeted lipidomics: a case study of peripheral blood mononuclear cells

Giovanni Ventura [1,*], Mariachiara Bianco [1,*], Cosima Damiana Calvano [1], Ilario Losito [1] and Tommaso R.I. Cataldi[1]

[1] Department of Chemistry, and Interdepartmental Research Center SMART University of Bari Aldo Moro, via Orabona 4, 70126, Bari, Italy

* Correspondence: giovanni.ventura@uniba.it & mariachiara.bianco@uniba.it

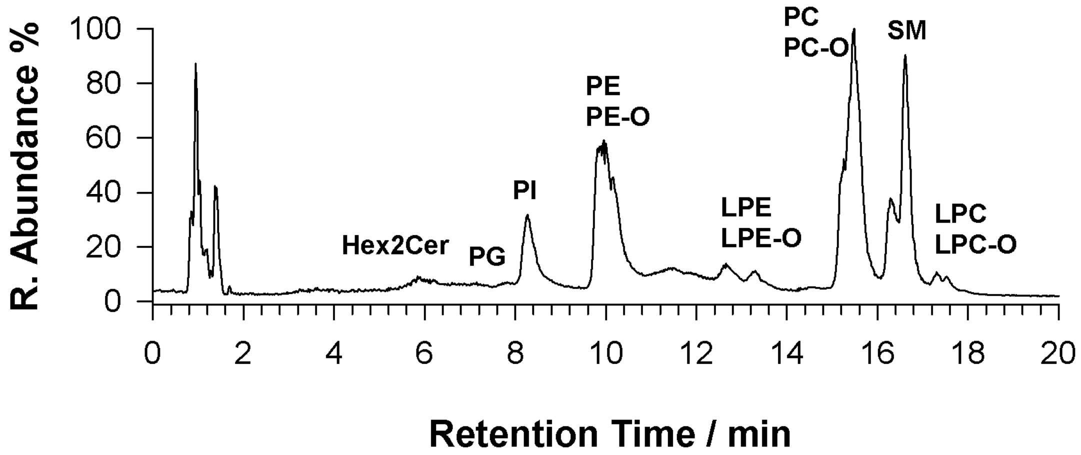

Figure S1. Representative total ion current (TIC) HILIC chromatogram acquired in negative ion mode of the analyzed PBMCs lipid extracts.

GPL Class	Diagnostic Prodduct Ions or Neutral Losses	
PCs, DMPEs<br>PC-O (*m/z* 224)<br>LPC X:Y/ 0 (I $_{m/z\ 224}$ > I $_{m/z\ 242}$)<br>LPC 0/X:Y (I $_{m/z\ 242}$ > I $_{m/z\ 224}$)	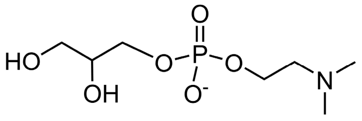<br>$C_7H_{17}NO_6P^-$ - *m/z:* 242.07990	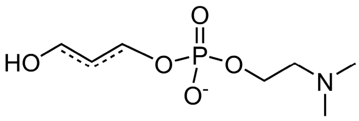<br>$C_7H_{15}NO_5P^-$ - *m/z:* 224.06933
PE<br>PE-O (*m/z* 196)<br>LPE X:Y/ 0 (I $_{m/z\ 196}$ > I $_{m/z\ 214}$)<br>LPE 0/X:Y (I $_{m/z\ 214}$ > I $_{m/z\ 196}$)	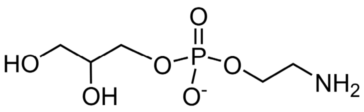<br>$C_5H_{13}NO_6P^-$ - *m/z:* 214.04860	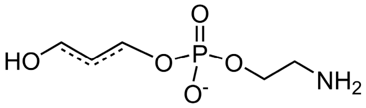<br>$C_5H_{11}NO_5P^-$ - *m/z:* 196.03803
PAs<br>PIs, PSs, PGs	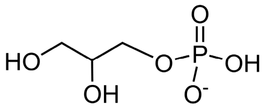<br>$C_3H_8O_6P^-$ - *m/z:* 171.00640	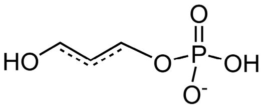<br>$C_3H_6O_5P^-$ - *m/z:* 152.99583
PIs	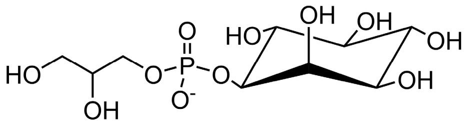<br>$C_9H_{18}O_{11}P^-$ (M^-) - *m/z:* 333.05922<br>$[M-H_2O]^-$ - *m/z:* 315.04866<br>$[M-2H_2O]^-$ - *m/z:* 297.03809	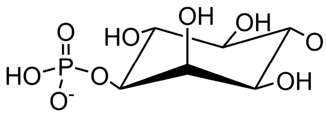<br>$C_6H_{12}O_9P^-$ (M^-) - *m/z:* 259.02244<br>$[M-H_2O]^-$ - *m/z:* 241.01188
PGs	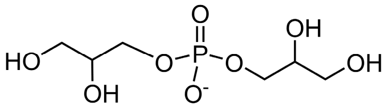<br>$C_6H_{14}O_8P^-$ (M^-) - *m/z:* 245.04318	$[M-H_2O]^-$ - *m/z:* 227.03261<br>$[M-2H_2O]^-$ - *m/z:* 209.02205<br>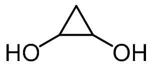 $C_3H_6O_2$ - 74.03678 Da
PSs	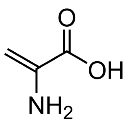	$C_3H_5NO_2$ - 87.03203 Da

Figure S2. Diagnostic product ions and neutral loss species for the most common GP classes. Calculated *m/z* or mass values, according to the case, are reported.

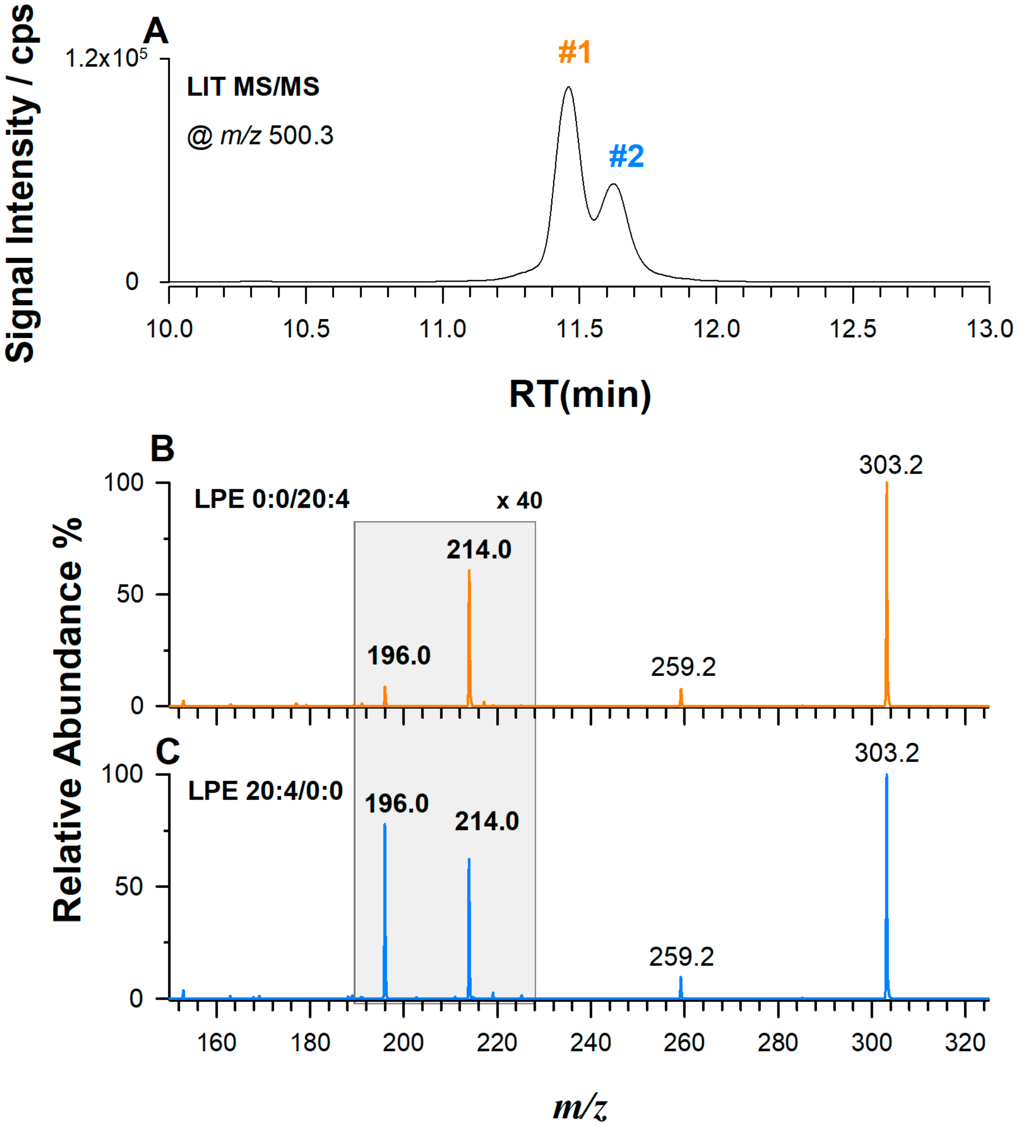

Figure S3. A) Chromatographic separation of LPE 20:4 regioisomers and MS/MS spectra averaged under the respective chromatographic peaks: B) LPE 0:0/20:4 and C) LPE 20:4/0:0.

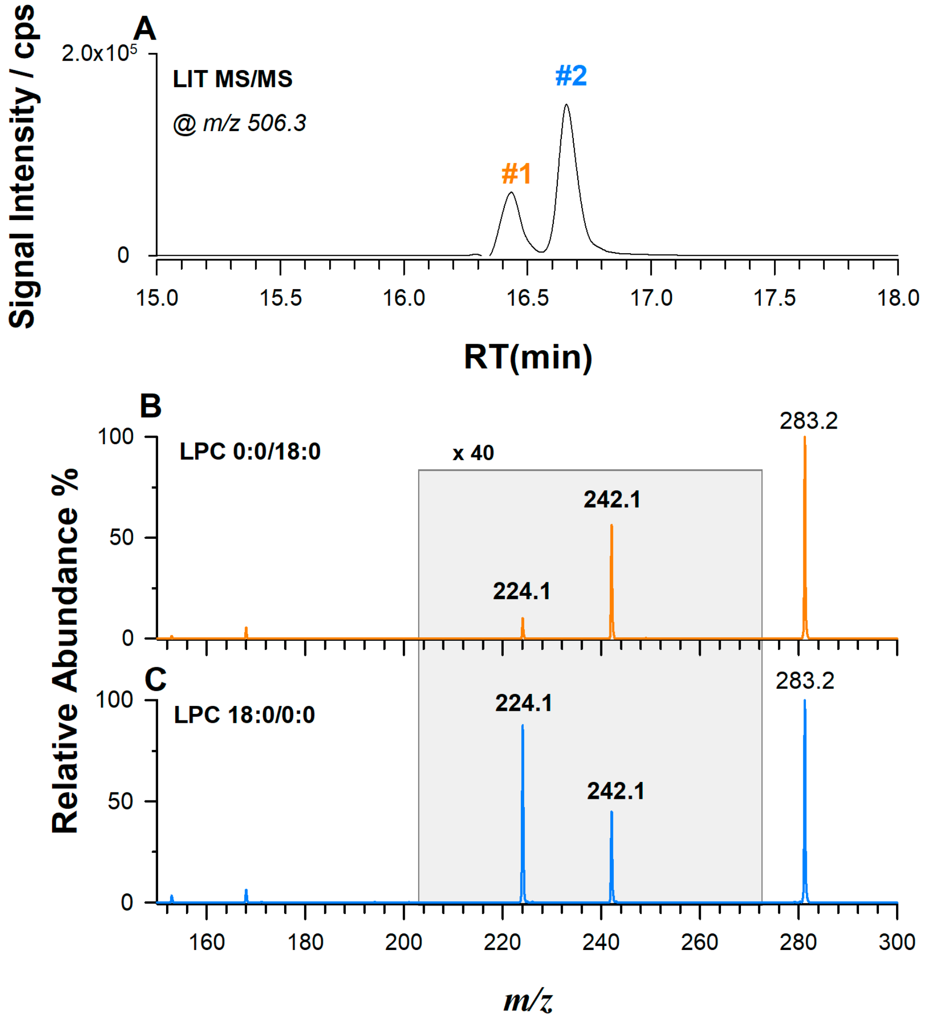

Figure S4. A) Chromatographic separation of LPC 18:0 regioisomers and MS/MS spectra averaged under the corresponding chromatographic peaks: B) LPC 0:0/18:0 and C) LPC 18:0/0:0

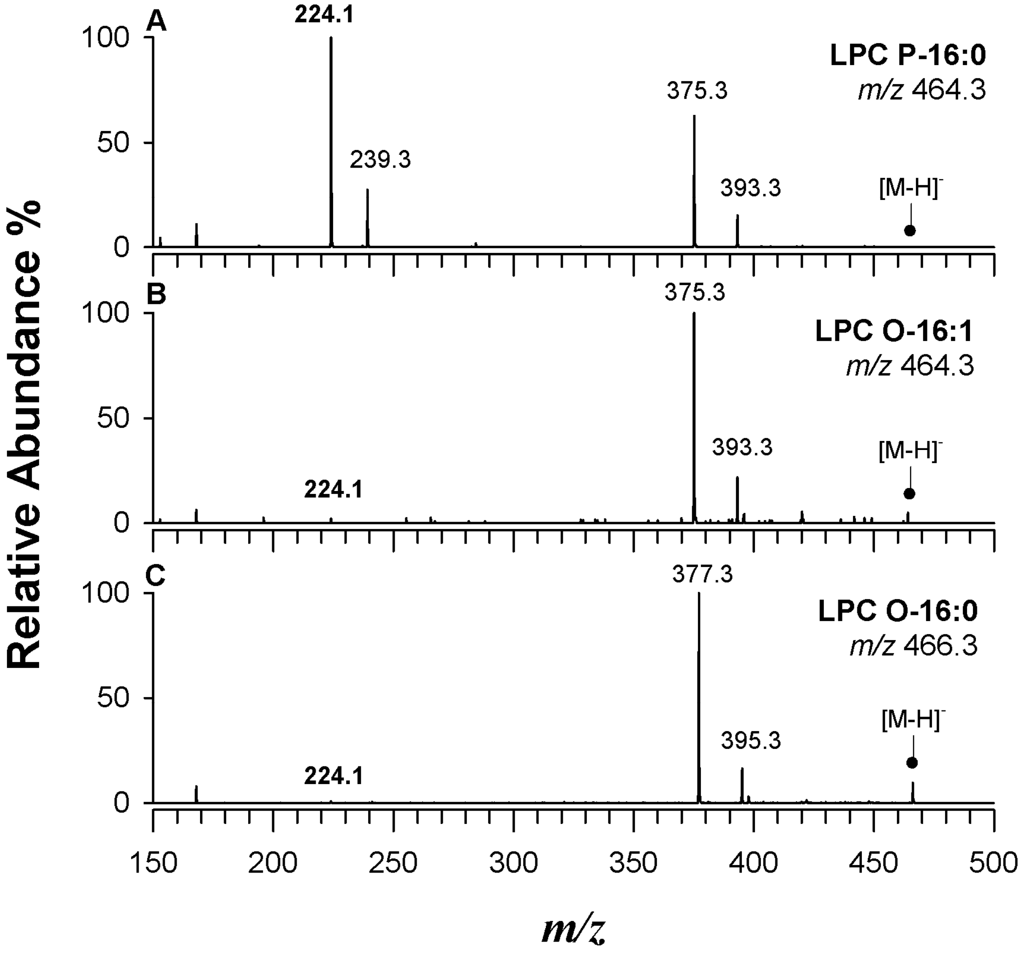

Figure S5. MS/MS spectra of A) LPC P-16:0 at *m*/*z* 464.3 B) LPC O-16:1 at *m*/*z* 464.3, and C) LPC O-16:0 at *m*/*z* 466.3.

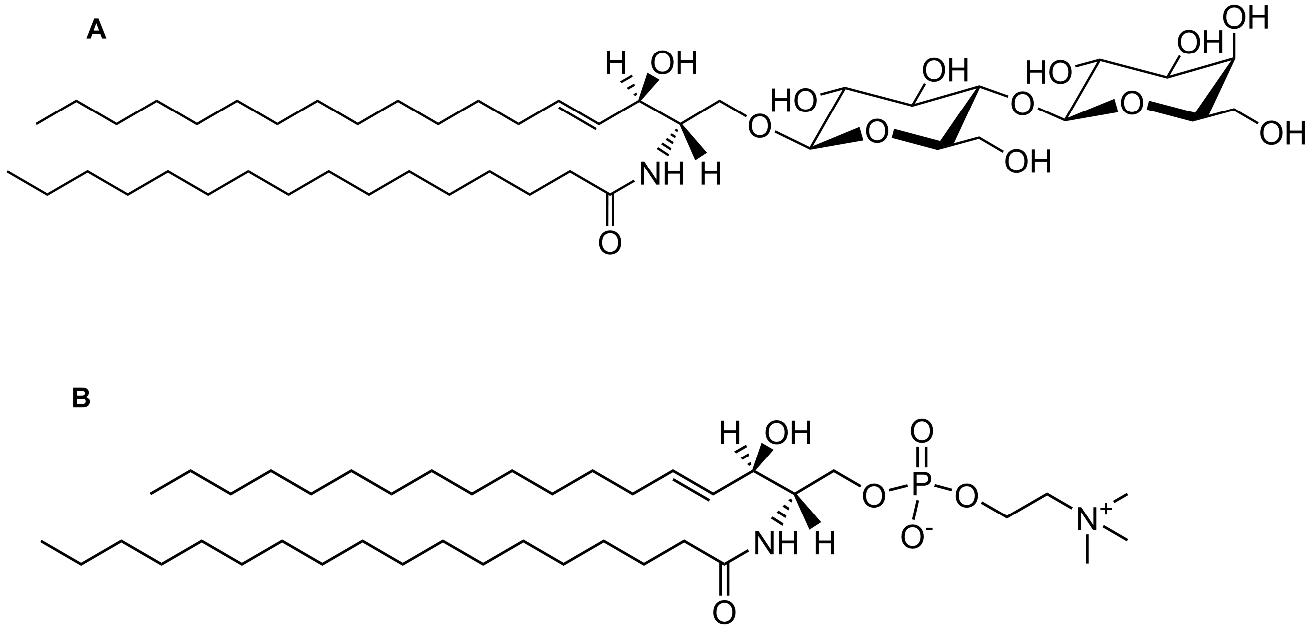

Figure S6. Structures of A) C16 Lactosyl(ß) Ceramide (d18:1/16:0), an Hex_2Cer, and B) SM (d18:1/18:1)

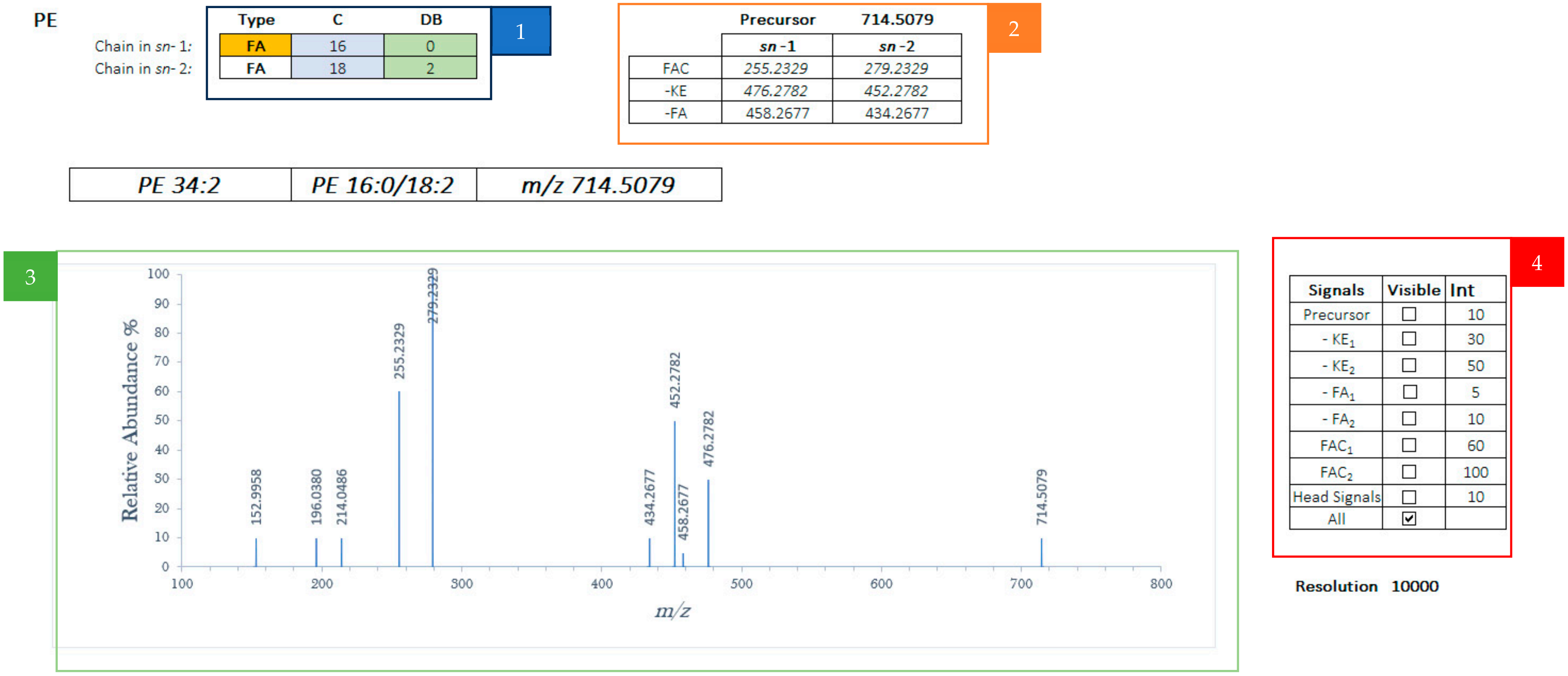

Figure S7. Excel-based tool for MS/MS lipid spectra simulation. (1) the composition of fatty acyl chains (FA X:Y) is entered; (2) summary table generates *m*/*z* values of expected ions; (3) the simulated spectrum is displayed. (4) relative intensities are adjusted based on experimental data. Checkboxes toggle ion visibility.

Table S1. PE and PC identified in human peripheral blood mononuclear cells.
Class normalized intensities are reported in **DOI: 10.1007/s00216-020-02817-z**

Class	*m/z*[a]	Specie	Assignment
PE	766.539	PE 38:4	PE 18:0/20:4
PE	742.539	PE 36:2	PE 18:1/18:1, PE 18:0/18:2
PE	744.554	PE 36:1	PE 18:0/18:1, PE 18:1/20:4, PE 16:0/22:5, PE 16:1/22:4
PE	716.524	PE 34:1	PE 16:0/18:1
PE	738.508	PE 36:4	PE 16:0/20:4
PE	794.571	PE 40:4	PE 18:0/22:4, PE 20:0/20:4, PE 20:2/20:2
PE	792.555	PE 40:5	PE 18:0/22:5, PE 20:1/20:4, PE 18:1/22:4
PE	740.523	PE 36:3	PE 18:1/18:2, PE 16:0/20:3
PE	714.508	PE 34:2	PE 16:0/18:2, PE 16:1/18:1
PE	790.539	PE 40:6	PE 18:0/22:6, PE 20:3/20:3
PE	762.508	PE 38:6	PE 16:0/22:6, PE 18:2/20:4, PE 16:1/22:5
PE	770.571	PE 38:2	PE 20:1/18:1, PE 20:2_18:0, PE 20:0/18:2
PE	788.524	PE 40:7	PE 20:4/20:3, PE 18:1/22:6
PE	752.524	PE 37:4	PE 17:0/20:4
PE	736.492	PE 36:5	PE 18:2/18:3, PE 16:1/20:4
PE	690.508	PE 32:0	PE 16:0/16:0
PE	782.571	PE 39:3	PE 21:3/18:0
PE	688.492	PE 32:1	PE 16:0/16:1
PE	754.539	PE 37:3	PE 19:2_18:1
PE	730.539	PE 35:1	PE 16:0/19:1, PE 17:0/18:1
PE	772.586	PE 38:1	PE 18:0/20:1
PE	786.508	PE 40:8	PE 20:4/20:4
PE	728.524	PE 35:2	PE 17:1_18:1
PE	820.586	PE 42:5	PE 22:1_20:4
PE	724.492	PE 35:4	PE 15:0_20:4
PE	756.555	PE 37:2	PE 19:1_18:1
PE	712.492	PE 34:3	PE 16:0/18:3
PE	818.571	PE 42:6	PE 22:4/20:4
PE	816.555	PE 42:7	PE 22:3_20:4
PE	726.508	PE 35:3	PE 17:1_18:2

PC	744.555	PC 34:1	PC 16:0/18:1
PC	718.539	PC 32:0	PC 16:0/16:0
PC	794.571	PC 38:4	PC 18:0/20:4, PC 20:3/20:4
PC	742.539	PC 34:2	PC 16:0/18:2, PC 16:1/18:1
PC	770.571	PC 36:2	PC 18:1/18:1, PC 18:0/18:2
PC	766.539	PC 36:4	PC 16:0/20:4
PC	772.586	PC 36:1	PC 18:0/18:1, PC 16:0_20:1
PC	768.555	PC 36:3	PC 18:1/18:2, PC 16:0/20:3
PC	792.555	PC 38:5	PC 18:1/20:4, PC 16:0_22:5, PC 18:2/20:3
PC	716.524	PC 32:1	PC 16:0/16:1, PC 14:0_18:1
PC	690.508	PC 30:0	PC 16:0/14:0, PC 15:0/15:0
PC	790.539	PC 38:6	PC 16:0/22:6, PC 18:2/20:4
PC	820.586	PC 40:5	PC 20:1/20:4, PC 18:0_22:5, PC 18:1_22:4
PC	818.571	PC 40:6	PC 18:0/22:6, PC 20:2/20:4, PC 18:1_22:5
PC	822.602	PC 40:4	PC 20:0/20:4, PC 18:0_22:4
PC	704.524	PC 31:0	PC 15:0/16:0
PC	816.555	PC 40:7	PC 20:3/20:4, PC 18:1/22:6
PC	798.602	PC 38:2	PC 20:1/18:1, PC 18:0_20:2
PC	758.571	PC 35:1	PC 16:0/19:1, PC 17:0/18:1
PC	732.555	PC 33:0	PC 17:0/16:0, PC 18:0_15:0
PC	730.539	PC 33:1	PC 17:1/16:0, PC 15:0_18:1
PC	740.524	PC 34:3	PC 16:0/18:3, PC 16:1/18:2
PC	814.539	PC 40:8	PC 20:4/20:4
PC	764.524	PC 36:5	PC 16:0/20:5, PC 16:1/20:4
PC	756.555	PC 35:2	PC 17:0_18:2, PC 19:2/16:0
PC	800.617	PC 38:1	PC 20:0_18:1, PC 20:1_18:0, PC 22:1/16:0
PC	714.508	PC 32:2	PC 14:0/18:2, PC 16:0/16:2, PC 16:1/16:1
PC	728.524	PC 33:2	PC 15:0_18:2
PC	842.571	PC 42:8	PC 22:4_20:4
PC	662.477	PC 28:0	PC 18:0_10:0, PC 14:0/14:0, PC 16:0_12:0
PC	688.492	PC 30:1	PC 16:0_14:1, PC 16:1_14:0

[a] For PCs, reported *m*/*z* refers to a demethylated adduct

Table S2. PC-O and PE-O identified in human peripheral blood mononuclear cells. Class normalized intensities are reported in DOI: 10.1007/s00216-020-02817-z

Class	*m/z*[a]	Species	Assignment
PC-O	730.576	PC-O 34:1	PC-O 18:1/16:0, PC O-16:0/18:1
PC-O	704.56	PC-O 32:0	PC O-16:0/16:0
PC-O	752.56	PC-O 36:4	PC O-16:0/20:4
PC-O	750.544	PC-O 36:5	PC-O 16:1/20:4
PC-O	780.591	PC-O 38:4	PC O-18:0/20:4, PC O-16:0/22:4
PC-O	728.56	PC-O 34:2	PC O-16:0/18:2, PC P-16:0/18:1
PC-O	732.591	PC-O 34:0	PC O-18:0/16:0, PC O-16:0/18:0
PC-O	702.544	PC-O 32:1	PC O-16:0/16:1
PC-O	756.591	PC-O 36:2	PC O-18:0/18:2, PC O-17:0/19:2
PC-O	754.576	PC-O 36:3	PC-O 18:1/18:2, PC O-16:0/20:3
PC-O	758.607	PC-O 36:1	PC O-18:0/18:1, PC-O 20:1/16:0
PC-O	676.529	PC-O 30:0	PC O-16:0/14:0, PC O-14:0/16:0
PC-O	808.623	PC-O 40:4	PC O-20:0/20:4, PC O-18:0/22:4
PC-O	760.623	PC-O 36:0	PC o-20:0/16:0
PE-O	750.544	PE-O 38:5	PE-O 18:1/20:4, PE-O 16:1/22:4
PE-O	722.513	PE-O 36:5	PE-O 16:1/20:4
PE-O	748.529	PE-O 38:6	PE-O 18:2/20:4, PE-O 16:1/22:5
PE-O	778.576	PE-O 40:5	PE-O 20:1/20:4, PE-O 18:1/22:4
PE-O	776.56	PE-O 40:6	PE-O 18:1/22:5, PE-O 18:2/22:4, PE-O 20:2/20:4
PE-O	774.544	PE-O 40:7	PE-O 18:1/22:6, PE-O 18:2/22:5, PE-O 20:3/20:4
PE-O	700.529	PE-O 34:2	PE O-16:0/18:2, PE P-18:1/16:0, PE P-18:0/16:1, PE P-16:0/18:1
PE-O	746.513	PE-O 38:7	PE-O 16:1/22:6, PE-O 18:3/20:4
PE-O	726.544	PE-O 36:3	PE-O 18:1/18:2, PE-O 18:2/18:1, PE P-16:1/20:2, PE O-16:0/20:3
PE-O	728.56	PE-O 36:2	PE-O 18:1/18:1, PE O-18:0/18:2, PE-O 16:1/20:1
PE-O	804.591	PE-O 42:6	PE-O 22:2/20:4, PE-O 20:1/22:5, PE-O 20:2/22:4, PE-O 18:1/24:5, PE-O 16:1/18:2
PE-O	772.529	PE-O 40:8	PE-O 18:2/22:6, PE-O 20:4/20:4
PE-O	806.607	PE-O 42:5	PE-O 22:1/20:4, PE-O 20:1/22:4
PE-O	802.576	PE-O 42:7	PE-O 20:1/22:6, PE-O 22:3/20:4
PE-O	720.497	PE-O 36:6	PE-O 16:1/20:5, PE-O 16:2/20:4
PE-O	702.544	PE-O 34:1	PE-O 18:1/16:0, PE O-16:0/18:1, PE-O 16:1/18:0
PE-O	756.591	PE-O 38:2	PE-O 20:1/18:1
PE-O	756.591	PE-O 38:2	PE-O 18:1/20:1
PE-O	674.513	PE-O 32:1	PE-O 16:1/16:0
PE-O	800.56	PE-O 42:8	PE-O 20:2/22:6, PE-O 22:4/20:4
PE-O	672.497	PE-O 32:2	PE-O 16:1/16:1
PE-O	730.576	PE-O 36:1	PE O-18:0/18:1
PE-O	730.576	PE-O 36:1	PE-O 20:1/16:0
PE-O	676.529	PE-O 32:0	PE O-16:0/16:0

[a] For PC-Os, reported *m/z* refers to a demethylated adduct

Table S3. PI, PG, and PS identified in human peripheral blood mononuclear cells. Accurate *m/z* values of deprotonated adducts, along with regiochemical assignment are provided. Class normalized intensities are reported in DOI: 10.1007/s00216-020-02817-z

Class	*m/z*	Specie	Assignment
PI	885.550	PI 38:4	PI 18:0/20:4
PI	887.566	PI 38:3	PI 18:0/20:3
PI	883.534	PI 38:5	PI 18:1/20:4
PI	857.519	PI 36:4	PI 16:0/20:4
PI	861.550	PI 36:2	PI 18:0/18:2, PI 18:1/18:1
PI	913.581	PI 40:4	PI 18:0/22:4, PI 20:0/20:4
PI	911.566	PI 40:5	PI 20:1/20:4, PI 18:0/22:5
PI	863.566	PI 36:1	PI 18:0/18:1
PI	909.550	PI 40:6	PI 20:2/20:4, PI 18:0/22:6
PI	859.534	PI 36:3	PI 18:1/18:2, PI 20:3/16:0
PI	899.566	PI 39:4	PI 19:0/20:4, PI 18:0/21:4
PI	871.534	PI 37:4	PI 17:0/20:4
PI	881.519	PI 38:6	PI 16:0_22:6, PI 20:4_18:2
PI	905.519	PI 40:8	PI 20:4/20:4
PI	809.519	PI 32:0	PI 16:0/16:0
PI	851.566	PI 35:0	PI 16:0_19:0
PG	747.518	PG 34:1	PG 18:1/16:0
PG	773.534	PG 36:2	PG 18:1/18:1, PG 18:0/18:2, PG 20:2/16:0, PG 20:1/16:1
PG	769.503	PG 36:4	PG 18:2/18:2, PG 20:4/16:0
PG	775.549	PG 36:1	PG 20:1/16:0, PG 18:0/18:1
PG	771.518	PG 36:3	PG 18:2_18:1, PG 20:3_16:0
PG	797.533	PG 38:4	PG 18:0_20:4
PG	761.534	PG 35:1	PG 19:1_16:0, PG 17:0_18:1
PG	795.518	PG 38:5	PG 18:1/20:4, PG 22:5/16:0
PG	745.502	PG 34:2	PG 18:2/16:0
PG	847.549	PG 42:7	PG 22:2_20:5
PG	869.659	PG 44:10	PG 22:5_22:5
PG	759.518	PG 35:2	PG 18:2_17:0
PG	721.503	PG 32:0	PG 16:0/16:0
PG	821.534	PG 40:6	PG 18:0_22:6
PG	771.518	PG 36:3	PG 18:2_18:1
PS	810.529	PS 38:4	PS 18:0/20:4
PS	788.545	PS 36:1	PS 18:0/18:1
PS	812.545	PS 38:3	PS 18:0/20:3
PS	786.529	PS 36:2	PS 18:1/18:1, PS 18:0/18:2
PS	832.513	PS 40:7	PS 20:4/20:3
PS	836.545	PS 40:5	PS 20:1/20:4, PS 18:0/22:5, PS 18:1/22:4
PS	814.560	PS 38:2	PS 18:1/20:1, PS 18:0/20:2
PS	816.576	PS 38:1	PS 18:1/20:0, PS 18:0/20:1

Table S4. Hex2Cer and SM identified in human peripheral blood mononuclear cells. Class normalized intensities are reported in DOI: 10.1007/s00216-020-02817-z

Class	*m/z*	Specie	Assignment [a]
Hex2Cer	860.587	Hex2Cer 34:1;O2	18:1;O2/16:0
Hex2Cer	970.696	Hex2Cer 42:2;O2	18:1;O2/24:1
Hex2Cer	944.681	Hex2Cer 40:1;O2	18:1;O2/22:0
Hex2Cer	972.712	Hex2Cer 42:1;O2	18:1;O2/24:0
Hex2Cer	916.65	Hex2Cer 38:1;O2	18:1;O2/20:0
Hex2Cer	942.665	Hex2Cer 40:2;O2	18:1;O2/22:1
Hex2Cer	958.697	Hex2Cer 41:1;O2	18:1;O2/23:0
Hex2Cer	888.618	Hex2Cer 36:1;O2	18:1;O2/18:0
Hex2Cer	832.556	Hex2Cer 32:1;O2	14:1;O2/18:0
Hex2Cer	858.572	Hex2Cer 34:2;O2	18:1;O2/16:0
SM	687.545 [b]	SM 34:1;O2	SM 18:1;O2/16:0
SM	771.639 [b]	SM 40:1;O2	SM 18:1;O2/22:0, SM 16:1;O2/24:0
SM	797.654 [b]	SM 42:2;O2	SM 18:1;O2/24:1
SM	743.607 [b]	SM 38:1;O2	SM 18:1;O2/20:0, SM 16:1;O2/22:0
SM	659.513 [b]	SM 32:1;O2	SM 18:1;O2/14:0, SM 16:1;O2/16:0
SM	715.576 [b]	SM 36:1;O2	SM 18:1;O2/18:0, SM 16:1;O2/20:0
SM	685.529 [b]	SM 34:2;O2	SM 18:2;O2/16:0
SM	799.670 [b]	SM 42:1;O2	SM 18:1;O2/24:0
SM	769.623 [b]	SM 40:2;O2	SM 18:1;O2/22:1, SM 16:1;O2/24:1
SM	713.560 [b]	SM 36:2;O2	SM 18:1;O2/18:1
SM	741.592 [b]	SM 38:2;O2	SM 18:1;O2/20:1
SM	757.623 [b]	SM 39:1;O2	SM 18:1;O2/21:0, SM 16:1;O2/23:0
SM	783.639 [b]	SM 41:2;O2	SM 18:1;O2/23:1

[a] The nomenclature (e.g., SM 18:1;O2/24:1) refers to a specific SL structure.
In this format, the first part (18:1;O2) indicates the number of C atoms, DBs, and O atoms in the sphingoid base, respectively, while the numbers following the slash denote the C atoms and DBs in the acyl chain. Note that the O count includes the O atom at position 1 of the ceramide.
[b] This *m/z* refers to a SM observed as a demethylated adduct
